# Development and validation of the HPLC–MS/MS method and its application to the pharmacokinetic study for the Mongolian drug Sendeng-4 in rat blood plasma

**DOI:** 10.3389/fphar.2025.1547415

**Published:** 2025-03-19

**Authors:** Pu Bai, Yu Dong

**Affiliations:** ^1^ Inner Mongolia Medical University, Hohhot, China; ^2^ Ordos School of Clinical Medicine, Inner Mongolia Medical University, Ordos, China; ^3^ Engineering Technology Research Center of Pharmacodynamic Substance and Quality Control of Mongolian Medicine in Inner Mongolia, Inner Mongolia Medical University, Hohhot, China

**Keywords:** Mongolian drug, Sendeng-4, myricetin, LC–MS/MS, pharmacokinetic, (2R, 3R)-dihydromyricetin

## Abstract

**Abstract:**

Sendeng-4 is a Mongolian drug. The Mongolian people have been using it to treat rheumatoid arthritis. At present, an increasing number of Han people are paying attention to the anti-rheumatoid effect of Sendeng-4. However, information on the pharmacokinetics of Sendeng-4 is limited, which limits its wide application in China.

**Objective:**

Liquid chromatography–tandem mass spectrometry (LC–MS/MS) was established to study the pharmacokinetics of Sendeng-4.

**Method:**

MS/MS with a negative ionization mode (ESI-) and multiple reaction monitoring at m/z 300.95→193.09 and 317.08→192.10 were detected for (2R, 3R)-dihydromyricetin and myricetin, respectively. The pharmacokinetic parameters were analyzed by DAS 2.0.

**Result:**

The results showed that the plasma concentration time (C–T) curves of (2R, 3R)-dihydromyricetin and myricetin showed double peaks. The T_max_ value of (2R, 3R)-dihydromyricetin and myricetin in both groups was 3 h. In absorption, the AUC_(0-∞)_ values of (2R, 3R)-dihydromyricetin and myricetin in the normal group and the arthritis model group were 16.151 ± 2.670 mg·h/L vs. 11.331 ± 0.749 mg·h/L and 2.626 ± 0.400 mg·h/L vs. 2.213 ± 0.388 mg·h/L, respectively. In the distribution, the Vz/F values of (2R, 3R)-dihydromyricetin and myricetin in the normal group and the arthritis model group were 8.212 L/kg vs. 1.744 L/kg and 5.252 L/kg vs. 10.568 L/kg, respectively. In metabolism, the MRT (0-∞) values of (2R, 3R)-dihydromyricetin and myricetin in the normal group and the arthritis model group were 6.848 h vs. 3.476 h and 5.661 h vs. 8.959 h, respectively. In excretion, the CLz/F values of (2R, 3R)-dihydromyricetin and myricetin in the normal group and the arthritis model group were 0.021 vs. 0.024 L/min/kg and 0.018 vs. 0.021 L/min/kg, respectively. There were significant variations in the absorption levels, distribution levels, and elimination rate of (2R, 3R)-dihydromyricetin and myricetin after the administration of Sendeng-4.

**Conclusion:**

The study laid the foundation for the subsequent study of pharmacokinetics of Sendeng-4 in humans. The results of this study will contribute to a better understanding of the activity and clinical application of Sendeng-4 and other related traditional Mongolian drug prescriptions.

## 1 Introduction

Rheumatoid arthritis (RA) is a common systemic autoimmune disease characterized by chronical, symmetrical, and erosive damage of the synovium of joints. Sendeng-4, a traditional Mongolian drug, is commonly used as an anti-rheumatic therapy in the clinic, which is recorded in “The People’s Republic of China Ministry of Health Standards for drug (Mongolian branch)” (National Pharmacopoeia Committee, 1998). This formula includes four herbs, namely, *Xanthoceras sorbifolium* Bunge (Yu et al., 2012; Wang et al., 2016) (Sapindaceae; Xanthoceras; *Xanthoceras sorbifolium* stem), *Melia azedarach* L (He et al., 2010; Liu et al., 2016; He et al., 2011) (Meliaceae; Melia; *Melia azedarach* fructus), *Gardenia jasminoides* (Lin et al., 2015; Sung et al., 2014) (Rubiaceae; Gardenia; *Gardenia jasminoides* fructus), and *Terminalia chebula* Retz (Manosroi et al., 2013; Eshwarappa et al., 2015) (Combretaceae; Terminalia; *Terminalia chebula* fructus).

**FIGURE 1:**
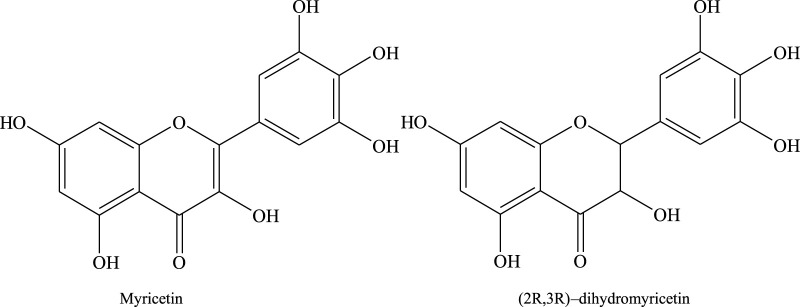
Structures of myricetin and (2R, 3R)-dihydromyricetin in Sendeng-4.

The Mongolian drug is a type of ethnic medicine and is a Chinese herbal medicine used by the Mongolian people in the Inner Mongolia region. The Mongolian drug is similar to traditional Chinese drugs. It consists of a variety of herbs, which contain complex chemical ingredients. Despite containing the same ingredients, it also shows different effects in different formulations, for example, (2R, 3R)-dihydromyricetin and myricetin (Figure 1) of Sendeng-4 in this study, which mainly have anti-inflammatory and antioxidant activities in the Sendeng-4 formula. According to the literature reported, (2R, 3R)-dihydromyricetin has anti-inflammatory, anti-oxidative, hypoglycemic, hepatoprotective, anti-browning, and antibacterial effects (Liang et al., 2014; Zhang et al., 2007), and myricetin has anti-inflammatory, anti-oxidative, antianaphylaxis, and anti-tumor effects (Li and Ding, 2012; Ko, 2012; Wu et al., 2016).

In pre-project studies, (2R, 3R)-dihydromyricetin and myricetin were isolated from Sendeng-4 in the extraction and separation experiment. In addition, through cell biological activity tests, it was proven that (2R, 3R)-dihydromyricetin and myricetin showed anti-inflammatory biological activity *in vitro*. We also established a HPLC–MS/MS method for simultaneously detecting the blood concentration of (2R, 3R)-dihydromyricetin and myricetin (Bai et al., 2020). Although plasma concentrations of (2R, 3R)-dihydromyricetin and myricetin were detected, the distribution, metabolism, and excretion of dihydromyricetin and myricetin in the body were not clear. Therefore, it is necessary to study the pharmacokinetic characteristics of (2R, 3R)-dihydromyricetin and myricetin. So, the established HPLC–MS/MS method (Bai et al., 2020) was applied to study the pharmacokinetics in normal and arthritis model rats.

## 2 Materials and methods

### 2.1 Chemicals and reagents

Myricetin (purity, >98%) was isolated in our laboratory (Department of Natural Medicinal Chemistry, Inner Mongolia Medical University, Hohhot, PR China), which was identified by combination NMR, mass spectrometry, and infrared spectroscopy, and purity was determined by HPLC chromatography. (2R, 3R)-Dihydromyricetin (purity, >98%) was purchased from J&K Scientific LTD. (Beijing, PR China), and the batch number is 110749–201316. Acetic acid was purchased from Sigma-Aldrich (St. Louis, MO, United States). Acetonitrile and methanol of HPLC grade were purchased from Fisher Scientific Co., Ltd (St. Louis, MO, United States). Ultrapure water was purified using a Milli-Q Reagent Water System (Millipore, Burlington, MA, United States). Collagen type II and Freund’s complete adjuvant were purchased from Sigma-Aldrich (St. Louis, MO, United States). All other chemicals and reagents used were of HPLC grade.

### 2.2 Animal and animal grouping

A total of 30 healthy Sprague–Dawley rats, weighing 220 ± 20 g, were purchased from the Experimental Animal Center of Inner Mongolia University (Hohhot, China). The rats were randomly divided into two groups: 15 rats in the normal group and 15 rats in the model group. Randomized grouping procedures are as follows: (1) Number: all rats were numbered from 1 to 30. (2) Obtain random numbers: 30 numbers were selected from rows 10 and columns 15 on the right side of the random number table to correspond to 30 numbered rats. (3) Find the remainder: 30 random numbers were divided by 2 to get the remainder. (4) Group: the rats corresponding to a random number with remainder 0 were assigned to the normal group. The rats corresponding to a random number with remainder 1 were assigned to the model group. The rats were kept in an air-conditioned animal room with a relative humidity of 50% ± 10% and temperature of 22°C ± 2°C. All rats had free access to water and food (the Experimental Animal Center of Inner Mongolia University, Hohhot, China). The rats were acclimatized to the environment for 15 days, and then, they were fasted for 12 h before the experiment. The animal studies were approved by the Animal Ethics Committee of Inner Mongolia Medical University (Hohhot, China).

### 2.3 Rat model of rheumatoid arthritis preparation

A model of collagen-induced arthritis was first reported by Trentham in 1977 (Myers et al., 2008). Based on the study of domestic and foreign experts and scholars over the years, this model is similar to human rheumatoid arthritis in pathology, immunology, and genetics (Griffiths et al., 1981; Wooley et al., 1981). So, it is recognized as an ideal animal model for screening and studying the treatment or prevention of rheumatoid arthritis drugs.

An emulsion (0.2 mL) made of Freund’s complete adjuvant and Collagen type II was injected into the hind feet of each rat. Twenty days later, the emulsion (0.1 mL) was injected at the same place of the rats. After two injections, the arthritis model was successfully induced.

### 2.4 Preparation of Sendeng-4 extracts

The *Xanthoceras sorbifolium* Bunge (Sapindaceae; Xanthoceras; *Xanthoceras sorbifolium* stem), *Melia azedarach* L (Meliaceae; Melia; *Melia azedarach* fructus), *Gardenia jasminoides* (Rubiaceae; Gardenia; *Gardenia jasminoides* fructus), and *Terminalia chebula* Retz (Combretaceae; Terminalia; *Terminalia chebula* fructus) samples were purchased from Inner Mongolia Hohhot Tianli Chinese herbal medicine company. These herbs were identified and authenticated by Professor Li Xiao in appearance, microscopic identification, and physical and chemical identification and stored in the Engineering Technology Research Center of Pharmacodynamic Substance and Quality Control of Mongolian Medicine in Inner Mongolia.

The four herbs were dried in the shade. In accordance with the proportion, the crude powder (2,000 g) was soaked in 70% ethanol (v/w, 10:1) for 1 h, followed by reflux extraction for 2 h. After filtration, the extracts were reflowed again in 70% ethanol (v/w, 10:1) for 2 h. The filtrate was pooled together and concentrated by a rotary evaporator to no alcohol at 45°C.

The solution was adsorbed by AB-8 macroporous resin and eluted with distilled water and ethanol. The ethanol elution was collected and concentrated to no alcohol. Finally, the dry extract powder of Sendeng-4 was obtained. The dry extract powder of Sendeng-4 (1.62 g) was weighed in a beaker. It was then dissolved in 0.5% CMC-Na (Qingyun et al., 2018) to acquire the Sendeng-4 extract solution at the concentration of 0.2025g/mL.

### 2.5 Preparation of blood samples

In this study, protein precipitation and liquid extraction were applied to extract (2R, 3R)-dihydromyricetin, myricetin, and IS from blood samples. A measure of 200 μL Plasma, 200 μL HCl (2 M), 50 μL 10% L-ascorbic acid, and 100 μL internal standard (IS) paeoniflorin solution (0.8 μg/μL) was added to a 5.0-mL Eppendorf tube. The mixture was vortex-mixed for 1 min at room temperature and placed for 30 min in an 80°C water bath. After 30 min, the sample was cooled to room temperature, 1 mL ethyl acetate was added, and centrifuged at 3,000 rpm for 10 min. Then, the supernatant was carefully removed and transferred to a new 1.5-mL Eppendorf tube and evaporated to dryness at 40°C under nitrogen gas. The residue was dissolved in 500 μL methanol−water (50:50, v/v). A portion of the supernatant (10 μL) was injected into the HPLC/MS/MS system for analysis.

### 2.6 Instruments and analytical conditions

The analysis was performed on a Thermo Finnigan Surveyor Plus HPLC tandem LCQ Advantage MAX Multi-Stage Ion Trap Mass Spectrometer (Thermo Fisher Scientific, Finnigan, United States). The high-performance liquid chromatography (HPLC) system consisted of a quaternionic pump (Thermo Fisher Scientific, United States), an autosampler (Thermo Fisher Scientific, United States), and a UV detector (Thermo Fisher Scientific, United States). LCQ Advantage MAX was equipped with an electrospray ionization (ESI) probe.

The specific parameters of the MS analysis are as follows: samples were detected by the multiple reaction monitoring (MRM) mode. The capillary voltage was 4.5 kv. The gas temperature was 250°C. The sheath gas was 30 L/min, and the aux/sweep gas flow rate was 5 L/min. The capillary temperature was 250°C.

The specific parameters of HPLC are as follows: the mobile phase consisted of acetonitrile (solvent A) and 0.1% acetic acid water (solvent B) at a flow rate of 1.0 mL/min. The gradient elution conditions are as follows: 0–5 min, 12%–14% A; 5–15 min, 14%–40% A, 15–20 min, and 40%–100% A. The injection volume was 10 μL. The detection wavelength was at 254 nm. The column temperature was 25°C.

We evaluated the following columns: Waters XBridge C-18 column (250 × 4.6 mm, 5.0 μm, Waters, Milford, United States), Extend ODS C-18 column (250 × 4.6 mm, 5.0 μm, Agilent, United States), Hypersil ODS-2 C-18 column (250 × 4.6 mm, 5.0 μm, Thermo Fisher Scientific, United States), and ShimNex CS C-18 column (250 × 4.6 mm, 5.0 μm, Shimaizumi, Japan). The column with the best separation was selected. Other instruments used are as follows: a SIGMA 3–18 K centrifugal machine, a HH-ZK4 Intelligent digital display thermostatic bath, and a G560E Vortex machine.

### 2.7 Stock solution, standard solution, and quality control sample solution preparation

The stock solutions of (2R, 3R)-dihydromyricetin and myricetin were prepared at the concentration of 71.8 ng/μL and 5.32 ng/μL, respectively. The standard solutions were obtained from the stock solution by dilution with methanol. The calibration sample solutions were prepared by adding 100 μL standard solutions to 200 μL blank plasma in 1.5 mL Eppendorf tubes. Therefore, the final calibration sample solutions were obtained at the concentrations of 0.399–39.900 ng/μL (2R, 3R)-dihydromyricetin and 0.050–5.026 ng/μL myricetin, respectively. The quality control (QC) sample solutions were prepared in the same way as the calibration samples.

The final prepared low-, medium-, and high-quality control concentrations of (2R, 3R)-dihydromyricetin were 0.399 ng/μL (LQC), 3.990 ng/μL (MQC), and 39.900 ng/μL (HQC), respectively. The concentrations of myricetin LQC, MQC, and HQC were 0.050 ng/μL, 0.503 ng/μL, and 5.026 ng/μL, respectively. The stock solution of the internal standard (IS), paeoniflorin, was prepared in methanol at the concentration of 2 mg/mL. The IS working solution of 0.8 μg/μL was obtained by diluting the stock solution with methanol.

### 2.8 Method validation

The method was validated in compliance with the FDA bioanalytical method validation guidelines (Fda, 2018).

#### 2.8.1 Specificity and sensitivity

The plasma chromatograms of (2R, 3R)-dihydromyricetin and myricetin were compared to determine whether the corresponding retention times of (2R, 3R)-dihydromyricetin, myricetin, and IS had impurity peaks and whether there was any interference between them.

#### 2.8.2 Linearity and LLOQ

The linear equation (y = ax + b) of (2R, 3R)-dihydromyricetin and myricetin was established by using the ratio of the peak area of (2R, 3R)-dihydromyricetin to the peak area of IS as the *y* value and the concentration of (2R, 3R)-dihydromyricetin and myricetin as the *x* value, and the linearity of the method of (2R, 3R)-dihydromyricetin and myricetin was verified. The lower limit of quantitation (LLOQ) value was established at the lowest concentration of the linear range, while the upper limit of quantification value was established at the highest concentration of the linear ranges.

#### 2.8.3 Precision and accuracy

The intra- and inter-day accuracy and precision were assessed by analyzing three levels of QC samples three times on three consecutive days. The accuracy and precision were evaluated as the relative error (RE) and the relative standard deviation (RSD), respectively. The RE was within ±15%, and the RSD could not exceed 15%.

#### 2.8.4 Extraction recovery and matrix effect

The extraction recovery and the matrix effect were evaluated by analyzing three levels of QC concentrations three times. The extraction recovery was calculated by comparing the plasma drug concentrations of plasma samples with (2R, 3R)-dihydromyricetin and myricetin added before extraction and with the equivalent amount of (2R, 3R)-dihydromyricetin and myricetin added after extraction. The matrix effect was evaluated by the post-extraction quantitative method. The matrix effect was calculated by comparing plasma drug concentrations of (2R, 3R)-dihydromyricetin and myricetin added to plasma samples after extraction with the plasma drug concentrations actually added.

#### 2.8.5 Stability

The stability was studied at different conditions analyzing three levels (LQC, MQC, and HQC) of QC sample solutions three times. The room temperature stability study analyzed the stability of the sample at room temperature over a period of 24 h. The freeze–thaw stability study was to detect the stability of samples after three freeze–thaw cycles at −75°C–25°C. The stability was evaluated by relative standard deviation (RSD). The RSD was within ±15%.

## 3 Application to a pharmacokinetic study

The pharmacokinetics of RA model rats and normal rats were investigated in the study. Rats were fed under standard environmental conditions (12 h light and 12 h dark cycle) for a few days before the pharmacokinetic study. After the rats were fasted for at least 12 h, they were orally administered (using the gavage technique) 5.4 g/kg Sendeng-4, which was suspended in 0.5% g/mL sodium carboxymethyl cellulose (CMC-Na). Under light anesthesia, blood samples (200 μL) were collected from orbital sinus at 0, 0.5, 1.0, 1.5, 2.0, 2.5, 3.0, 4.0, 5.0, 6.0, 7.0, and 8.0 h after oral administration. Plasma samples were centrifuged at 12,000 rpm for 10 min. The concentrations of (2R, 3R)-dihydromyricetin and myricetin were measured by the developed LC–MS/MS method. The parameters of pharmacokinetics (t_1/2_, t_max_, and C_max_) were calculated by DAS 2.0.

The C–T curve can reflect the qualitative characteristics of the pharmacokinetics, while the area under the curve (AUC), mean residence time (MRT), maximal plasma concentration (C_max_), and T1/2 can quantitatively reflect the systemic exposure level of the drug. The PK results of (2R, 3R)-dihydromyricetin and myricetin of Sendeng-4 were analyzed and discussed from the above two aspects.

## 4 Statistical analysis

DAS 2.0 software (Mathematical Pharmacology Professional Committee of China, Shanghai, China) was used for the pharmacokinetic studies. All data were shown as mean ± standard deviation (SD).

## 5 Results

### 5.1 Method development

**FIGURE 2:**
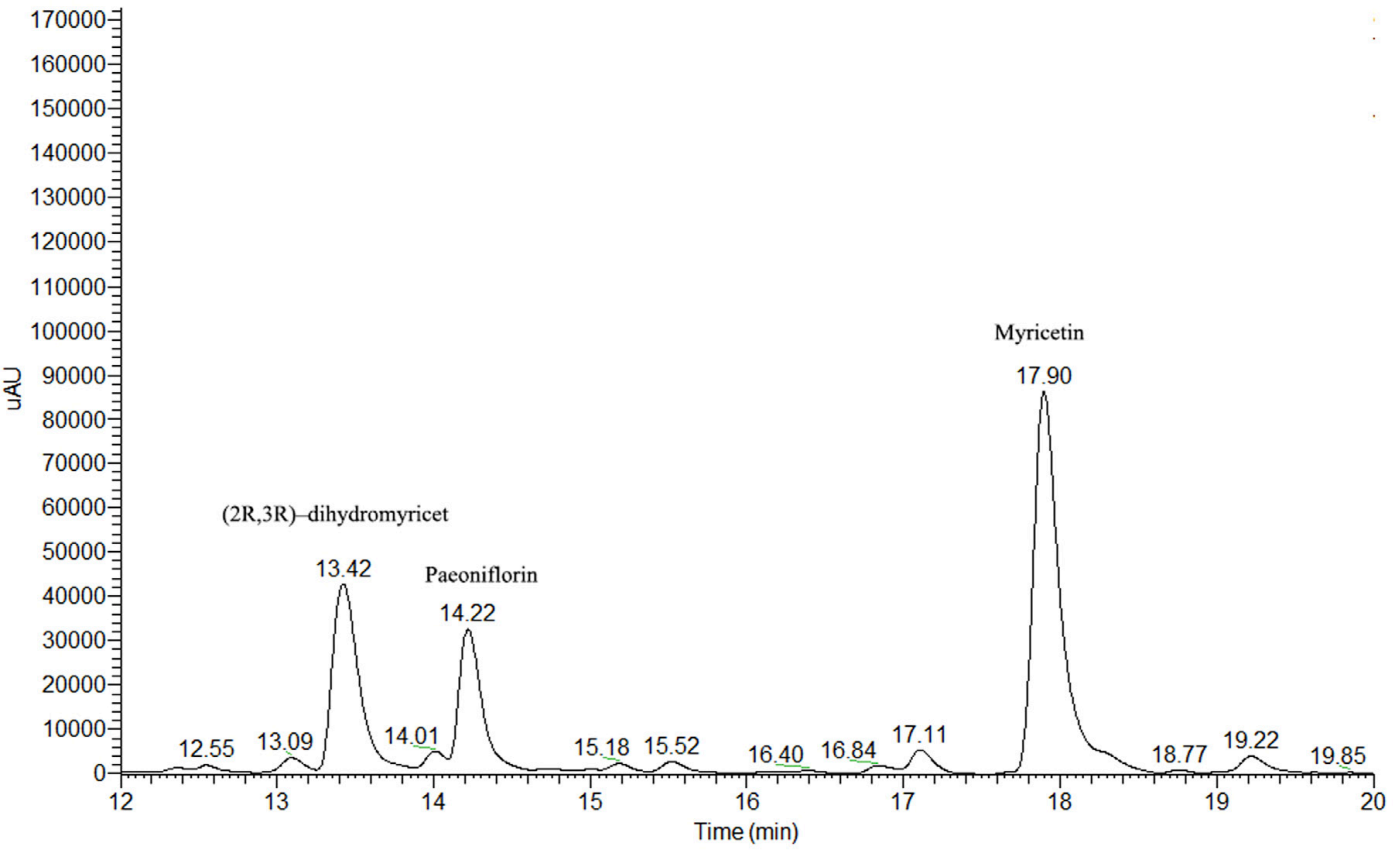
Chromatography of myricetin, (2R, 3R)-dihydromyricetin, and IS in rat blood plasma.

In terms of HPLC column selection, several types of HPLC columns were evaluated, such as Agilent Extend ODS C-18, Thermo Hypersil ODS-2 C-18, SHIMADZU ShimNex CS C-18, and Waters XBridge C-18 column. The data revealed that the Waters C-18 column exhibited better separation of peaks. Thus, the Waters C-18 column was chosen. During the HPLC run time of 20 min, (2R, 3R)-dihydromyricetin, myricetin, and IS (paeoniflorin) were well-separated with retention times of 13.42, 14.22, and 17.90 min, respectively (Figure 2).

The rat blood plasma samples were pretreated by organic solvent precipitation protein method. A variety of organic solvents were evaluated, including acetone, methanol, acetonitrile, and trichloroacetic acid, among others. The matrix effect of acetonitrile was the lowest, and the recovery rate of acetonitrile was the highest. So, we chose acetonitrile protein precipitation.

### 5.2 Mass spectral study of myricetin and (2R, 3R)-dihydromyricetin

**SCHEME 1:**
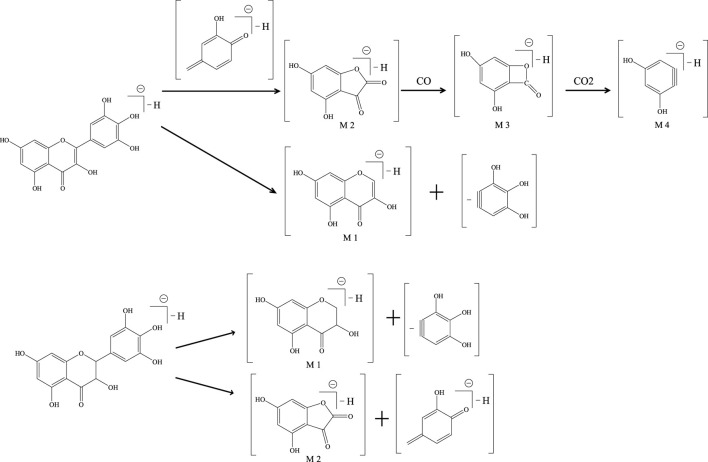
Mass spectrum cracking pathway of myricetin and (2R, 3R)-dihydromyricetin.

**FIGURE 3:**
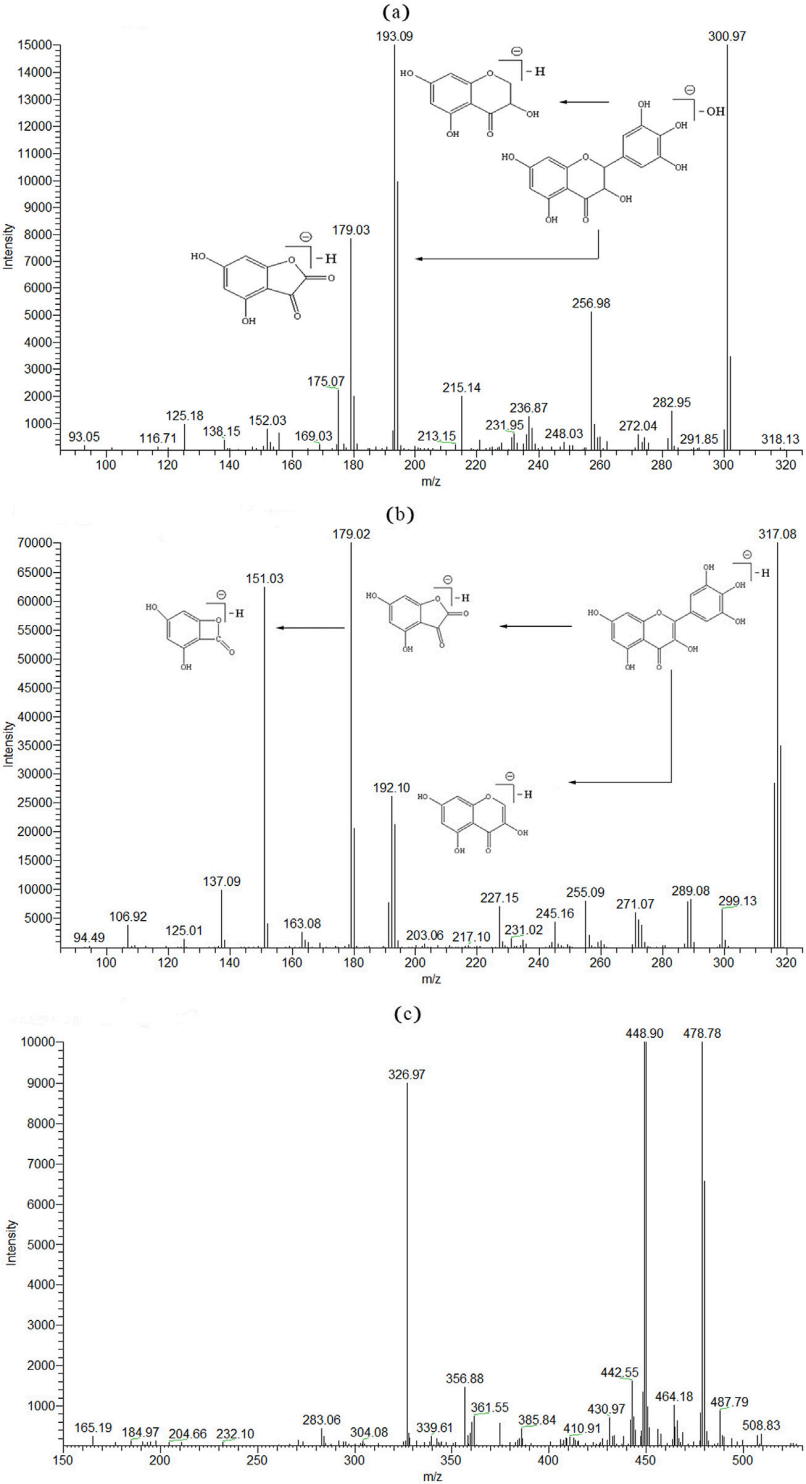
MS/MS spectrum of (2R, 3R)-dihydromyricetin **(A)**, myricetin **(B)**, and paeoniflorin **(C)**.

The mass spectrometry detection conditions were optimized and modified. The full ion mode scanning procedure was used to find the most optimum precursor-to-product ion pairs. Both positive and negative ionization ESI modes were tested for (2R, 3R)-dihydromyricetin, myricetin, and paeoniflorin (IS). The negative mode (ESI-) of (2R, 3R)-dihydromyricetin and myricetin had better sensitivity than the positive mode. (2R, 3R)-dihydromyricetin and myricetin were detected and quantified in the negative ionization mode (ESI-). The selective ion monitoring mode was used for quantitation by the [M−H]^−^ molecular ions of the analytes (Table 1). Under ionic bombardment, (2R, 3R)-dihydromyricetin and myricetin were split into different fragment ions through a cleavage reaction (Scheme 1) (Fabre et al., 2001). Among them, (2R, 3R)-dihydromyricetin and myricetin showed the same m/z 179.02 characteristic MS fragment in the MS spectra due to the similar molecular structure (Figure 3; Table 2).

**TABLE 1:** Molecular formula and fragment information on myricetin, (2R, 3R)-dihydromyricetin, and paeoniflorin (IS).

Analyte	Molecular formula	Molecular weight	Pathway	MS (*m/z*)	Fragment (*m/z*)	Retention time (min)
(2R, 3R)-Dihydromyricetin	C_15_H_12_O_8_	320	[M−H]−	319	300.95, 256.98, 193.09, and 179.03	10.58
Myricetin	C_15_H_10_O_8_	318	[M−H]−	317	317.08, 192.10, 179.02, 151.03, 137.09, 125.01, and 106.92	15.23
Paeoniflorin (IS)	C_23_H_28_O_11_	480	[M−H]−	479	478.84, 448.90, and 326.97	11.71

**TABLE 2:** Mass spectral data on myricetin, (2R, 3R)-dihydromyricetin, and its fragmentation.

Flavonol type and flavanone type	Molecular formula	Precursor ion (*m/z*, [M−H]^−^)		Product ions (*m/z*)
		Calculated	Observed	
Myricetin				
[M−H]^−^	C_15_H_10_O_8_	317	317.08	—
M1 (M-C_6_H_4_O_3_)	C_9_H_5_O_5_	192	192.10	124
M2 (M-C_7_H_5_O_2_)	C_8_H_3_O_5_	179	179.02	109
M3 (M2-CO)	C_7_H_3_O_4_	151	151.03	28
M4 (M3-CO_2_)	C_6_H_3_O_2_	107	106.92	44
(2R, 3R)-Dihydromyricetin				
[M−H_2_O]^−^	C_15_H_9_O_7_	301	300.95	18
M1 (M-C_6_H_4_O_3_)	C_9_H_5_O_5_	193	193.09	124
M2 (M-C_7_H_6_O_2_)	C_8_H_3_O_5_	179	179.03	109

### 5.3 Method validation

#### 5.3.1 Specificity and selectivity

**FIGURE 4:**
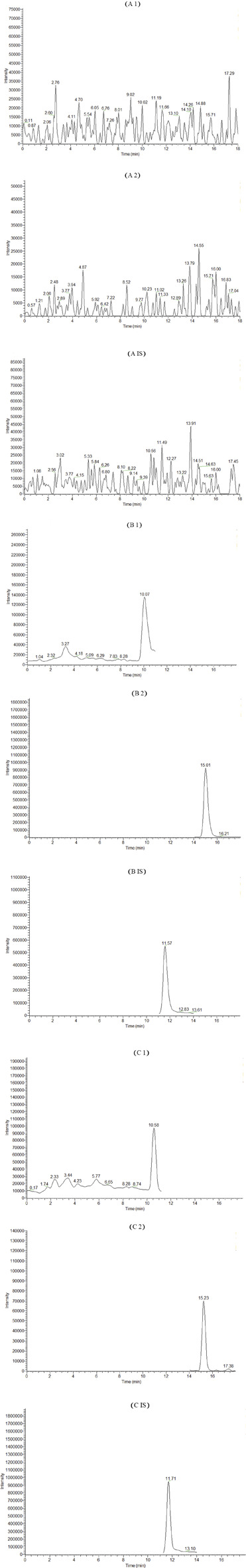
Extracted ion chromatogram (EIC) of the two ingredients and paeoniflorin (IS) in rats. **(A)** Blank plasma. **(B)** Blank plasma spiked with standard substances. 1: myricetin, 2: (2R, 3R)-dihydromyricetin, and IS: paeoniflorin. **(C)** Rat blood plasma after oral administration of Sendeng-4.

The chromatograms of rat blank blood plasma, rat blank blood plasma spiked with standard solutions and IS, and rat blood plasma after oral Sendeng-4 administration are shown in Figure 4. The retention times of myricetin, (2R, 3R)-dihydromyricetin, and the IS (paeoniflorin) were 15.23 min, 10.58 min, and 11.71 min, respectively. By comparison with the chromatogram of rat blank plasma, it was found that the endogenous substance did not interfere with (2R, 3R)-dihydromyricetin, myricetin, and IS. These results showed that the method developed has good selectivity without endogenous substance interferences.

#### 5.3.2 Linearity and lower limit of quantitation (LLOQ)

The calibration curves for (2R, 3R)-dihydromyricetin and myricetin exhibited good linearity, with coefficients of correlation (r) within the range of 0.9998–0.9999. Linear ranges, slopes, intercepts, LLOQs, and correlation coefficients of calibration curves are listed in Table 3. The correlation coefficients (r) were more than 0.9999. It indicated that our method has good linearity.

**TABLE 3:** Calibration curves, linear ranges, correlation coefficients, and LLOQ of (2R, 3R)-dihydromyricetin and myricetin.

Analyte	Linear regression equation	R^2^	Linear range (ng/μL)	LLOQ (ng/μL)
(2R, 3R)-Dihydromyricetin	y = 2E-05x+0.0083	0.9998	0.399–39.900	0.199
Myricetin	y = 0.0004x-0.0052	0.9997	0.050–5.026	0.025

#### 5.3.3 Precision and accuracy

The intra-day and inter-day precision and accuracy were evaluated three times. The results are summarized in Table 4. The relative standard deviations of intra- and inter-day were less than 15%. These results showed that the precision and accuracy was accurate and reliable.

**TABLE 4:** Precision and accuracy of (2R, 3R)-dihydromyricetin and myricetin in rat blood plasma.

Analyte	Spiked concentration (ng/μL)	Calculated concentration (ng/μL)	Intra-day precision (RSD, %)	Accuracy (RE, %)	Calculated concentration (ng/μL)	Inter-day precision (RSD, %)	Accuracy (RE, %)
(2R,3R)-Dihydromyricetin	0.399	0.375 ± 0.017	4.530	6.047	0.387 ± 0.012	3.005	3.058
	3.990	3.941 ± 0.126	3.185	1.277	3.873 ± 0.273	7.062	2.935
	39.900	41.874 ± 0.831	1.983	4.947	39.580 ± 1.088	2.749	0.803
Myricetin	0.050	0.050 ± 0.002	3.444	0.514	0.050 ± 0.003	6.601	0.095
	0.503	0.542 ± 0.010	1.906	7.671	0.482 ± 0.036	7.392	4.084
	5.026	5.149 ± 0.257	4.997	2.457	4.928 ± 0.344	6.979	1.943

#### 5.3.4 Extraction recovery and matrix effect

The extraction recovery and matrix effect of (2R, 3R)-dihydromyricetin and myricetin are shown in Table 5. The extraction recovery of (2R, 3R)-dihydromyricetin and myricetin were within the range of 91.609%–110.180% and 92.111%–103.933%, respectively. The matrix effect of (2R, 3R)-dihydromyricetin and myricetin were within the range of 94.289%–99.919% and 97.091%–99.820%, respectively. The results indicated that endogenous substances did not have a significant effect on the quantification of all analytes.

**TABLE 5:** Recovery of (2R, 3R)-dihydromyricetin and myricetin in rat blood plasma.

Analyte	Spiked concentration (ng/μL)	Extraction recovery		Matrix effect	
		Mean ± SD (%)	RSD (%)	Mean ± SD (%)	RSD (%)
(2R, 3R)-Dihydromyricetin	0.399	93.939 ± 4.684	4.986	99.919 ± 1.523	1.524
	3.990	110.180 ± 3.487	3.164	94.289 ± 3.281	3.480
	39.900	91.609 ± 5.470	5.971	99.051 ± 4.780	4.826
Myricetin	0.050	93.921 ± 2.443	2.601	97.933 ± 5.886	6.011
	0.503	92.111 ± 1.652	1.793	97.091 ± 2.743	2.825
	5.026	103.933 ± 4.806	4.624	99.820 ± 0.907	0.908

#### 5.3.5 Stability

As shown in Table 6, the relative standard deviations (RSDs) of room temperature stability and freeze–thaw stability were within 15% ((2R, 3R)-dihydromyricetin: <3.287% vs. < 2.865 and myricetin: <7.709 vs. < 5.275). These results indicated that (2R, 3R)-dihydromyricetin and myricetin were stable and applicable.

**TABLE 6:** Stability of (2R, 3R)-dihydromyricetin and myricetin in rat blood plasma under various storage conditions.

Analyte	Spiked concentration (ng/μL)	Room temperature stability	Freeze–thaw stability
		RSD (%)	RSD (%)
(2R, 3R)-Dihydromyricetin	0.399	3.287	1.281
	3.990	2.904	2.865
	39.900	3.257	2.847
Myricetin	0.050	1.909	5.275
	0.503	7.709	2.144
	5.026	3.577	1.048

### 5.4 Results of pharmacokinetic (PK) studies

DAS 2.0 software was used to fit the data to the compartmental model, and the AIC values of different models were compared. The one-compartment model, two-compartment model, three-compartment model, and different weights were used to fit the curve of the data, and the results showed that the one-compartment model with non-intravenous injection (weight 1/cc) had a good fit with the measured blood concentration, and the AIC value of the fitted model was the lowest.

#### 5.4.1 Pharmacokinetics of Sendeng-4 in normal rats

**FIGURE 5:**
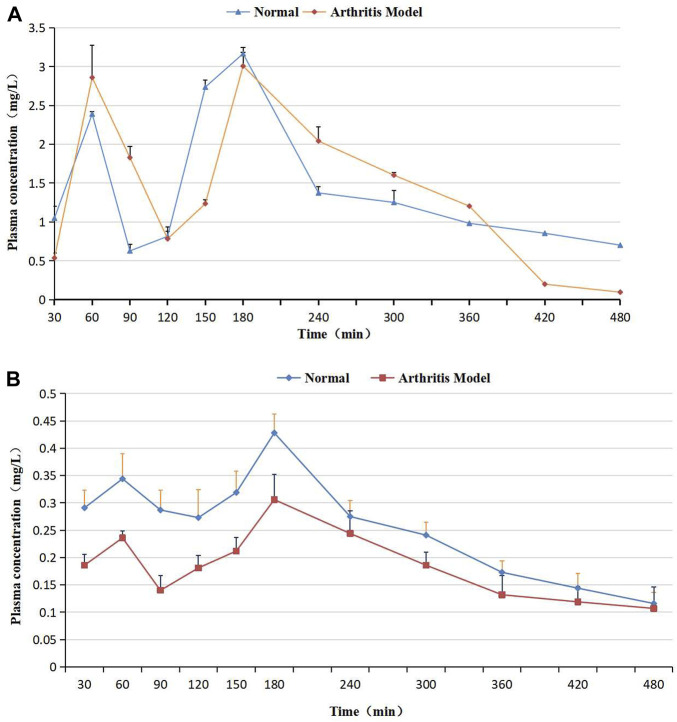
**(A)** Plasma concentration–time curves of (2R, 3R)-dihydromyricetin after oral Sendeng-4 administration in normal and arthritis model rats. **(B)** Plasma concentration–time curves of myricetin after oral Sendeng-4 administration in normal and arthritis model rats.

The plasma C-T curves of (2R, 3R)-dihydromyricetin and myricetin after oral Sendeng-4 administration are shown in Figure 5, and the corresponding PK parameters are listed in Table 7.

**TABLE 7:** Pharmacokinetic parameters of (2R, 3R)-dihydromyricetin and myricetin after oral administration of Sendeng-4 in normal and arthritis model rats.

Parameter		(2R, 3R)-Dihydromyricetin		Myricetin	
		Normal	Arthritis model	Normal	Arthritis model
C_max_	mg/L	3.166 ± 0.083	3.507 ± 0.174	0.428 ± 0.084	0.306 ± 0.046
T_max_	h	3	3	3	3
T_1/2z_	h	4.686	0.843	3.556	5.948
AUC_(0→t)_	mg·h/L	10.938 ± 0.096	11.219 ± 0.752	1.971 ± 0.221	1.386 ± 0.259
AUC_(0-∞)_	mg·h/L	16.151 ± 2.670	11.331 ± 0.749	2.626 ± 0.400	2.213 ± 0.388
MRT_(0-t)_	h	3.630	3.414	3.511	3.818
MRT_(0-∞)_	h	6.848	3.476	5.661	8.959
CLz/F	L/min/kg	0.021	0.024	0.018	0.021
Vz/F	L/kg	8.212	1.744	5.252	10.568

In Figure 5, the C–T curve showed a double peak after oral Sendeng-4 administration. Two plasma concentration peaks were observed at 1h and 3 h in the C–T curves of (2R, 3R)-dihydromyricetin and myricetin, and the maximum plasma concentration peak was at 3 h. The C–T curve approximates the one-compartment model (calculated by using DAS 2.0 software, Table 7). In absorption, the AUC_(0-∞)_ values of (2R, 3R)-dihydromyricetin and myricetin were 16.151 ± 2.670 mg·h/L and 2.626 ± 0.400 mg·h/L, respectively. In distribution, the Vz/F of (2R, 3R)-dihydromyricetin and myricetin were 8.212 L/kg and 5.252 L/kg, respectively. In metabolism, the MRT_(0-∞)_ of (2R, 3R)-dihydromyricetin and myricetin were 6.848 h and 5.661 h, respectively. In discharge, the CLz/F values of (2R, 3R)-dihydromyricetin and myricetin were 0.021 L/min/kg and 0.018 L/min/kg, respectively.

#### 5.4.2 Pharmacokinetics of Sendeng-4 in arthritis-induced rats

The arthritis-induced rats’ plasma C–T curves of (2R, 3R)-dihydromyricetin and myricetin are shown in Figure 5, and the corresponding PK parameters are listed in Table 7. Similarly, the C–T curves showed double peaks after oral Sendeng-4 administration in Figure 5, and the C–T curves approximated the one-compartment model (calculated by using DAS 2.0 software, Table 7). Two plasma concentration peaks were observed at 1h and 3 h in the C–T curves of (2R, 3R)-dihydromyricetin and myricetin. The T_max_ values of (2R, 3R)-dihydromyricetin and myricetin in both groups was 3 h. In absorption, the AUC_(0-∞)_ of (2R, 3R)-dihydromyricetin and myricetin were 11.331 ± 0.749 mg·h/L and 2.213 ± 0.388 mg·h/L, respectively. In distribution, the Vz/F values of (2R, 3R)-dihydromyricetin and myricetin were 1.744 L/kg and 10.568 L/kg, respectively. In metabolism, the MRT_(0-∞)_ values of (2R, 3R)-dihydromyricetin and myricetin were 3.476 h and 8.959 h, respectively. In discharge, the CLz/F values of (2R, 3R)-dihydromyricetin and myricetin were 0.024 L/min/kg and 0.021 L/min/kg, respectively.

## 6 Discussion

Comparing other traditional botanical drug therapies across the world, the most important characteristic of the Mongolian drug is the multi-herbs treatment based on the theory and unique treatment mode of Mongolian medicine. Due to the interaction of various ingredients in the Mongolian drug, the pharmacokinetic study of the Mongolian drug is complicated.

Through the study of network pharmacology, it was found that (Tian and Dong, 2015) (2R, 3R)-dihydromyricetin and myricetin, respectively, act on tumor necrosis factor-α and nuclear transcription factor Kappa B, and xanthine dehydrogenase/oxidase exerts anti-inflammatory effects. It is proven that Sendeng-4, a Mongolian drug, has a treatment effect on rheumatoid arthritis. Pre-project studies of cell biological activity tests proved that (2R, 3R)–dihydromyricetin and myricetin are active ingredients of Sendeng-4.

The LC–MS method was used to detect the drug concentration in biological samples with the advantages of good selectivity and high sensitivity. At present, it is increasingly used in the detection of pharmacokinetics and drug concentrations (Fang et al., 2016; Tao et al., 2013; Huisi et al., 2016; Qian et al., 2017; Tong et al., 2006; Yu et al., 2007). However, individual differences in rats, drug dissolution, and biological sample processing methods make it difficult to detect the tested ingredients. The concentration of the ingredients detected by the LC–MS/MS technique used the selected ion monitoring mode scan and molecular ion ionization mode.

As shown in Figure 5, the plasma C–T curves of (2R, 3R)-dihydromyricetin and myricetin showed double peaks. The reasons for the double peaks were summarized as follows: (a) Enterohepatic circulation (Okusanya et al., 2007): after oral administration, the drug entered into the liver through the portal vein, and a part of the drug accumulated in the gallbladder. When the gallbladder contracted, this part of the drug was rapidly released to the intestinal tract and reabsorbed. If the reabsorption of the drug is large enough to cause two increases in blood concentration, the C–T curve appears as double peaks. (b) Time of gastric emptying: the drug reached the small intestine two times. This causes the drug to enter the blood two times. Double peaks come into being in C–T curves (Chang and Shojaei, 2004; Metsugi et al., 2008). (c) Multiple site absorption in the gastrointestinal tract: there are multiple absorption sites in different parts of the gastrointestinal tract. However, the permeability of different sites in the inner membrane is different. Therefore, the absorption time and absorption rate of different parts were not consistent after oral administration, and double peaks came into being in C–T curves (Lennernäs and Regårdh, 1993; Piyapolrungroj et al., 2000). (d) Multi ingredient characteristics of the traditional Chinese drug. The multi-peak phenomenon of pharmacokinetics is increasingly complex. This is closely related to the multi-ingredient characteristics of the traditional Chinese drug. Since most traditional Chinese drugs contain a large number of ingredients with the same parent nucleus, the ingredients easily transform each other under environmental conditions in the body. In the mutual transformation of ingredients, the concentration of some ingredients increases and results in a multi-peak phenomenon.

## 7 Conclusion

This study established a simple, rapid, and stable HPLC–MS/MS method. The LC–MS/MS method satisfies all of the validation criteria suggested in the bioanalytical method validation guidelines from the FDA (Food and Drug Administration, 2020) and proves the specificity, reliability, and repeatability of the established quantitative method. This method improves the specificity and sensitivity of detection. In addition, the pretreatment method is simple with the high recovery rate, and the endogenous substances and impurities in the biological samples have no interference with the determination of substance. This method is applicable to pharmacokinetic studies of the Mongolian drug Sendeng-4 in rat blood plasma. The plasma concentration of normal rats was higher than that of arthritis model rats. There were significant differences in the absorption level, distribution level, and elimination rate of (2R, 3R)-dihydromyricetin and myricetin between arthritis model rats and normal rats. This result indicates that arthritic rats significantly consume (2R, 3R)-dihydromyricetin and myricetin, which indirectly proves that (2R, 3R)-dihydromyricetin and myricetin are active ingredients of Sendeng-4. The study lays the foundation for the subsequent study of pharmacokinetics of Sendeng-4 in humans and contributes to the wide application of Sendeng-4 in clinical practice.

## Data Availability

The raw data supporting the conclusions of this article will be made available by the authors, without undue reservation.
